# The impact of burnout syndrome among medical students - meta-analysis

**DOI:** 10.1192/j.eurpsy.2022.2215

**Published:** 2022-09-01

**Authors:** L. Hogea, L. Corsaro

**Affiliations:** 1 “Victor Babes” University of Medicine and Pharmacy, Department Of Neurosciences, Timisoara, Romania; 2 Campus Bio Medico, Plastic Surgery, Roma, Italy

**Keywords:** medical students, burnout syndrome, self-accomplishment

## Abstract

**Introduction:**

The medical profession is associated with high requirements and responsibilities, and high rates of burnout have been reported in the medical literature.

**Objectives:**

The aim of this study was to provide a detailed systematic review, focused on the impact of occupational burnout syndrome among medical students. A detailed perspective of existing instruments which are the psychometric properties and a meta-analysis of the average values of those three subscales of the most commonly applied tool - Maslach Burnout Inventory (MBI-HSS).

**Methods:**

The meta-analysis was performed based on the available data on burnout rates in medical students measured by the Maslach Burnout Inventory (MBI-HSS) method. In order to define the eligibility criteria for finding the relevant literature, the PICO method - the “population-intervention-comparison-result” approach was used.

**Results:**

The sample sizes included ranged from n = 73 to 4050 students. Mean values (M) ranged from 12.94 to 28.26 for emotional exhaustion and from 7.30 to 13.43 for depersonalization. M for personal achievement ranged from 31.3 to 38.07. Weighted averages and standard deviations were M = 22.93 (SD = 10.25) for emotional exhaustion, M = 8.88 (SD = 5.64) for depersonalization, and M = 35.11 (SD = 8.03) for self-accomplishment. The included studies reported different prevalence rates with burnout rates ranging from 7.0% to 75.2%. The prevalence rate of burnout measured by MBI-HSS varied between 10.0% and 63.4%.

**Conclusions:**

The meta-analytical aggregation of eligible studies showed high values of “emotional exhaustion”, “depersonalization” and “self-accomplishment”.

**Disclosure:**

No significant relationships.

